# Gastrointestinal adverse events of metformin treatment in patients with type 2 diabetes mellitus: a systematic review and meta-analysis with meta-regression of observational studies

**DOI:** 10.1186/s12902-024-01727-w

**Published:** 2024-09-30

**Authors:** Katarzyna Nabrdalik, Mirela Hendel, Krzysztof Irlik, Hanna Kwiendacz, Igor Łoniewski, Tommaso Bucci, Uazman Alam, Gregory Y. H. Lip, Janusz Gumprecht, Karolina Skonieczna-Żydecka

**Affiliations:** 1 Department of Internal Medicine, Diabetology and Nephrology, Faculty of Medical Sciences in Zabrze, Medical University of Silesia, 3 May Street, Zabrze, Katowice, 41-800 Poland; 2 Liverpool Centre for Cardiovascular Science at University of Liverpool, Liverpool John Moores University and Liverpool Heart & Chest Hospital, Liverpool, UK; 3 Students’ Scientific Association By the Department of Internal Medicine, Diabetology and Nephrology in Zabrze, Faculty of Medical Sciences in Zabrze, Medical University of Silesia, Katowice, Poland; 4 Department of Biochemical Science, Pomeranian Medical University, Szczecin, Poland; 5 Department of General and Specialized Surgery, Sapienza University of Rome, Rome, Italy; 6 Diabetes & Endocrinology Research and Pain Research Institute, Institute of Life Course and Medical Sciences, University of Liverpool and Liverpool University Hospital NHS Foundation Trust, Liverpool, UK; 7 Centre for Biomechanics and Rehabilitation Technologies, Staffordshire University, Stoke-On-Trent, UK; 8 Department of Clinical Medicine, Aalborg University, Aalborg, Denmark

**Keywords:** Gastrointestinal adverse events, Diarrhea, Dose, Formulation, Meta-analysis, Metformin

## Abstract

**Introduction:**

Metformin is the most prescribed medication for type 2 diabetes mellitus (T2DM); there is a well-established link with the elevated incidence of gastrointestinal (GI) adverse events (AE) limiting its administration or intensification.

**Objectives:**

The objective of this systematic review and meta-analysis of observational studies was to evaluate the pooled incidence of GI AE related to metformin use in patients with T2DM.

**Materials and methods:**

PUB MED/CINAHL/Web of Science/Scopus were searched from database inception until 29.07.2024 for observational studies in English describing the frequency of GI AE in patients with T2DM treated with metformin. Random-effects meta-analyses were used to derive effect sizes: event rates.

**Results:**

From 7019 publications, we identified 211 potentially eligible full-text articles. Ultimately, 21 observational studies were included in the meta-analysis. The prevalence of GI AE was as follows: diarrhea 6.9% (95% CI: 0.038–0.123), bloating 6,2% (95% CI: 0.020–0.177), abdominal pain 5,3% (95% CI: 0.003–0.529), vomiting 2.4% (95%: CI 0.007–0.075), constipation 1.1% (95%: CI 0.001–0.100). The incidence of bloating (coefficient -4.46; *p* < 0.001), diarrhea (coefficient -1.17; *p* = 0.0951) abdominal pain (coefficient -2.80; *p* = 0.001), constipation (coefficient -5.78; *p* = 0.0014) and vomiting (coefficient -2.47; *p* < 0.001) were lower for extended release (XR) metformin than metformin immediate release (IR) formulation.

**Conclusions:**

This study highlights the prevalence of GI AE in patients receiving metformin, with a diarrhea predominance, followed by bloating, diarrhea, abdominal pain, constipation, and vomiting. The incidence is lower in patients administered with XR metformin.

**Trial registration:**

https://www.crd.york.ac.uk/prospero/display_record.php?ID=CRD42021289975, identifier CRD42021289975.

**Supplementary Information:**

The online version contains supplementary material available at 10.1186/s12902-024-01727-w.

## Introduction

Metformin, derived from biguanide, has been in extensive use for therapy of type 2 diabetes mellitus (T2DM) for almost seven decades [1]. In 2005 the International Diabetes Federation (IDF) recommended metformin, as a first-line treatment for T2DM [2] and remained in this position until 2023 [3]. In 2018 American Diabetes Association (ADA) and European Association for the Study of Diabetes (EASD) introduced the concept of patient-centered care recognizing other new glucose-lowering drugs which could be the first choice of therapy when cardiovascular (CV) and renal co-morbidities or CV risk factors are present [4]. According to the newest ADA and EASD recommendations, pharmacologic strategies that provide sufficient efficacy to achieve and sustain treatment goals, such as metformin or other medications combination therapy, should be taken into consideration when starting glucose lowering therapy [5]. Nevertheless, the use of metformin is common and the new glucose-lowering drugs are usually evaluated as an add-on to an existing metformin therapy as a standard procedure [6–9]. Moreover, metformin is a well-studied drug, with many positive pleiotropic properties [10–14] and due to its affordable price [15] and good metabolic control it remains popular in many countries all over the world.

However, metformin administration may be linked to the side effects limiting its use, specifically GI related ones, which are common and were found to affect up to 20%-30% of patients of which approximately 5% discontinued the treatment which was evaluated in a study conducted 20 years ago [16]. In our recent meta-analysis and meta-regression of randomized controlled trials (RCT), we showed that the risk of GI AE such as abdominal pain, nausea and diarrhea was significantly higher in T2DM patients treated with metformin compared to other glucose-lowering drugs or placebo [17].

Despite the very widespread clinical use of metformin, there is a lack of systematic evidence regarding the risk of GI AE of the drug, with the exception of our recent meta-analysis of RCTs where we compared metformin to other glucose-lowering drugs or placebo [17] and other meta-analyses, and systematic reviews comparing different metformin formulations [16, 18] and network meta-analyses that focused mainly on drugs other than metformin [19, 20].

The presented study reports the frequency of GI AE in observational studies related to metformin treatment in patients with T2DM.

## Materials and methods

The protocol for this systematic review, meta-analysis, and meta-regression has been registered in the International Prospective Register of Systematic Reviews (Prospero), under the registration number CRD42021289975. The review was conducted in accordance with the Preferred Reporting Items for Systematic Reviews and Meta-Analysis (PRISMA) guidelines. This investigation builds upon our earlier research, which systematically evaluated the risk of GI AE risk in RCTs among patients with T2DM treated with metformin [17].

### Study selection

We confined our investigation to observational studies which enrolled patients with T2DM who were treated with metformin at any dosage, without adjunctive glucose-lowering medications, across any range of health outcomes. The principal aim was to quantify the prevalence of GI AE in this population. Assessed GI AE encompassed abdominal pain, bloating, constipation, diarrhea, nausea, vomiting, and the frequency of ceasing therapy due to AE. Two authors (KI, MH) independently conducted the initial screening process, which involved title and abstract review. Discrepancies in eligibility were resolved through consultation with a clinical leader (KN). The screening of full-text articles was also carried out independently by two authors (KI, MH). For reduplication purposes, the Zotero reference manager was employed. After the electronic search, a manual review of the reference lists of relevant systematic reviews was done.

### Data extraction

Data were extracted by two independent reviewers (KI and MH) and included key attributes such as study design, geographic location, and funding sources. The two investigators also abstracted study cohorts for age, sex, body mass index (BMI), average fasting blood glucose (FBG), average postprandial blood glucose (PBG), average HbA1c and any existing comorbidities. Additionally, the specific type of metformin formulation (IR or XR) dosage, and history of prior metformin use were assessed.

### Outcomes

Co-primary outcomes were the rates of the following: i) abdominal pain, ii) bloating, iii) constipation, iv) diarrhea, v) nausea, vi) vomiting.

### Risk of bias assessment

The first reviewer (HK) independently evaluated the risk of bias in the included studies using the Newcastle–Ottawa Scale (NOS). When disagreements arose, a second reviewer (IŁ) was consulted for adjudication. A study was classified as high quality if received a score of at least 7 points.

### Data and resource availability

Details pertaining to the search strategy, as well as inclusion and exclusion criteria, can be found in the Supplementary Material. The structured database with extracted data is available on request.

### Data synthesis and statistical analysis

We conducted a random effects meta-analysis of outcomes for which ≥ 2 studies contributed data, using Comprehensive Meta-Analysis V4 (http://www.meta-analysis.com). The between-study variance (τ2) was estimated using the method of moments (DerSimonian and Laird) and [21] the assumption of homogeneity in effects was tested using the Q statistic with k-1 degree of freedom (k – the number of studies). For nominal outcomes, the event rate was calculated. A two-tailed Z test was used to test the null hypothesis that the event rate was zero. In addition to classical meta-analysis, a meta-regression was performed under the random-effects model for both continuous and nominal study level covariates. The regression models with single covariates were fit. Meta-regression variables included: i) dosage of metformin (continuous moderator), ii) type of metformin (IR vs. XR) used (categorical moderator), iii) preexisting metformin treatment (categorical moderator), iv) average age (continuous moderator), v) gender (male %), (categorical moderator), vi) average BMI (continuous moderator), vii) average FBG (continuous moderator), viii) average PBG (continuous moderator), ix) average HbA1c (continuous moderator). Finally, we inspected funnel plots and used Egger’s regression test and the Duval and Tweedie’s trim and fill method, if necessary, to quantify whether publication bias could have influenced the results [22, 23]. All analyses were two-tailed with alpha = 0.05.

### Ethics

The study did not require ethical approval.

## Results

### Search results

**Fig. 1:**
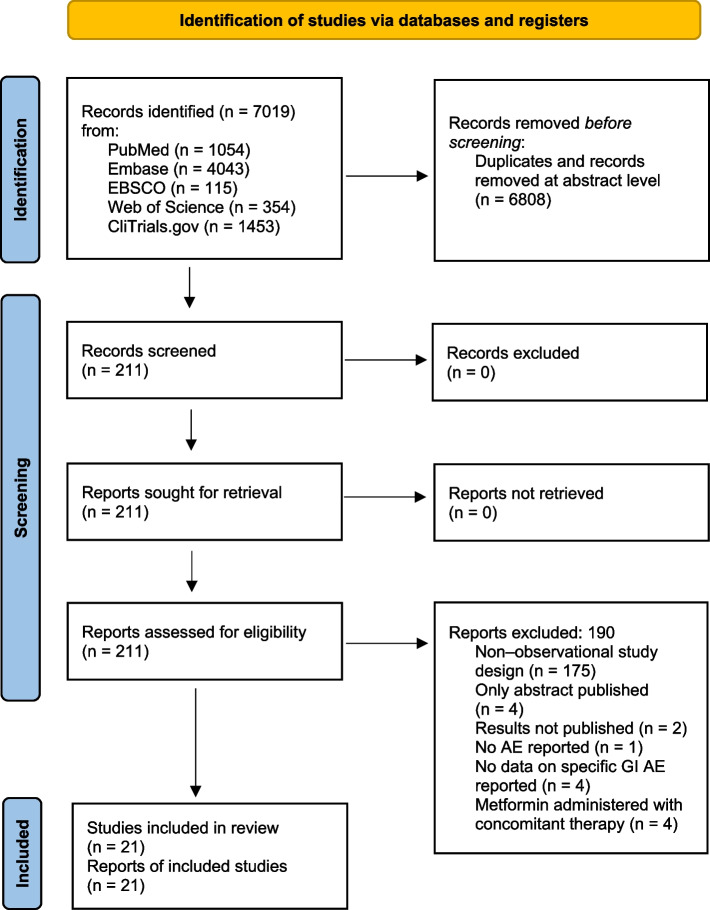
Study flow chart

The initial search yielded 7019 hits. There were 6808 studies excluded as duplicates and/or after evaluation at the title/abstract level. There were no additional studies identified via hand search. Eventually, 211 full-text articles were reviewed. Of those**,** 190 were excluded due to not fitting inclusion criteria. The reasons for exclusion are presented in Fig. 1. At last, 21 studies were included in the meta-analysis.

### Study and studied subjects' characteristics

In total, 21 studies including the number of *n* = 25206 patients that were treated with metformin were included into the final synthesis. Studies were mostly monocentric and carried out across various ethnic populations. Both male and female patients were included, with the mean presence of males equal to 45.13%. The median age of study participants was 57.4 years. Data on individual studies and participants’ characteristics are summarized in Table 1.

**Table 1:** Study and studied subjects’ characteristics

**Study characteristics**		**MET treatment**				**Patient characteristics**					
**Reference**	**Aim of study**	**total analyzed** **(n)**	**preexisting or started (P/S)**	**dosage (mg/day)**	**treatment form XR/IR**	**age (mean ± SD)**	**male** **(n/%)**	**BMI at baseline (kg/m**^**2**^**)**	**FBG (mg/dl)** **(mean ± SD)**	**PBG** **(mg/dl)** **(mean ± SD)**	**HbA1c** **(%)** **(mean ± SD)**
Aladhab et al., 2023 [24]	To detect the association of the OCT1 genetic polymorphism with the efficacy and GI AE of metformin in newly diagnosed T2DM and drug naïve patients	102	S	1000	IR	52.24 ± 10.74	54/52.9	NR	NR	NR	NR
Alibrahim et al., 2023 [25]	To study the effect of variables that may influence the development of metformin side effects and/or intolerance	148	NR	2000	NR	49.1 ± 6.6	72/48.6	31.89	175.83 ± 36.86	235.99 ± 102.46	9.55 ± 1.46
Al-Waeli et al. 2022 [26]	To determine the barriers interfering with optimal metformin dosage	475	NR	729,789	NR	56.2 ± 9.9	201/42.3	30.6	196.73 ± 79.87	260.73 ± 101.77	9.89 ± 2.65
Asche et al., 2008 [27]	Evaluation of AE of metformin, sulfonylureas and thiazolidinediones in geriatric patients	5438	S	NR	NR	72.2 ± 5	977/42	31.4	NR	NR	7.5 ± 1.5
Dandona et al., 1983 [28]	To determine the prevalence of diarrhea in biguanide treated patients	285	P	NR	NR	NR	NR	NR	NR	NR	NR
Das et al., 2021 [29]	To evaluate the clinical characteristics, treatment patterns, and clinical effectiveness and safety of high doses of metformin in Indian adults with T2DM	5695	P	2500	NR	50 ± 2.43	3480/62.7	27.7	114.75 ± 4.86	176.25 ± 8	NR
De Jong et al., 2016 [30]	Frequency, latency time, outcome and management of AE related to the use of metformin	2490	S	NR	NR	59.2 ± 10.2	1478/59.4	29.6	NR	NR	NR
Florez et al., 2010 [31]	Impact of metformin on GI symptoms and HRQL and metformin-associated GI AE in patients with T2DM newly beginning therapy	360	S	NR	NR	67 ± 15.5	140/38.9	NR	NR	NR	NR
Huang et al., 2015 [21]	An assessment of whether Helicobacter pylori infection could influence on metformin tolerance in patients with T2DM	415	S	1089	IR	64 ± 11.6	228/54.9	25.34	132.5 ± 42.8	NR	8.4 ± 2
Kim et al., 2012 [22]	Tolerability and antihyperglycemic efficacy of metformin XR in the treatment of patients with T2DM from six Asian countries	3556	S	840	XR	57.2 ± 11.5	1727/47.6	25.27	199.6 ± 63.7	NR	8.04 ± 1.35
Levy et al., 2010 [23]	The efficacy, tolerability and patient satisfaction of an XR formulation of metformin in patients with T2DM	61	P	1500	XR	54.1 ± 12.1	21/34.4	31.18	NR	NR	7.6 ± 1.5
Malik et al., 2023 [32]	To evaluate efficacy and safety of various oral antidiabetic drugs/regimens used for T2DM patients	248	NR	NR	NR	NR	140/56.4	NR	135.1 ± 13.2	193.12 ± 20	7.82 ± 0.6
Memon et al., 2022 [33]	To analyze and compare the pharmacological efficacy of sitagliptin and metformin in terms of blood glucose and glycated HbA1c T2DM patients	100	NR	NR	NR	49.7 ± 6.5	NR	NR	235.9 ± 32.5	421.5 ± 27.5	12.5 ± 2.7
Mishra et al., 2021 [34]	To monitor AE of anti-diabetic medication in OPD of tertiary care hospital of northern India	120	NR	NR	NR	NR	68/56.7	NR	NR	NR	NR
Okayasu et al., 2012 [35]	Evaluation of AE and further analyzed risk factors in Japanese patients with T2DM who initially administered metformin	101	S	621	IR	60.7 ± 14.86	62/61.4	24.7	NR	NR	9.4 ± 2.2
Raičević et al., 2023 [36]	To investigate possible risk factors for the occurrence of GI complaints in patients on MTF therapy	330	S	1000	NR	64 ± 14.7	141/42.7	26.64	NR	NR	NR
Riyaz et al., 2014 [37]	To compere sitagliptin to metformin as an initial monotherapy in patients with T2DM	100	NR	2000	IR	NR	NR	NR	NR	NR	7.95 ± NR
Sadeeqa et al., 2019 [38]	To investigate the effect of metformin-induced GI problems and its prevalence	300	P	673	IR	NR	77/33.9	NR	NR	NR	NR
Strojek et al., 2016 [39]	To assess adherence and tolerability of metformin XR formulation in patients with T2DM	4737	P	1667	XR	60.6 ± 9.4	2269/47.9	30.5	122 ± 24	152 ± 32	7.05 ± 0.86
Sumitani et al., 2012 [40]	Effectiveness of metformin and lifestyle interventions as an initial treatment in Japanese patients with newly diagnosed T2DM	23	S	1435	IR	53 ± 11	20/87	25.7	182 ± 59	NR	9.1 ± 2.1
Umamaheswaran et al., 2015 [41]	The impact of SLC22A1 rs622342 gene polymorphism on the clinical efficacy of metformin in South Indian T2DM patients	122	S	2250	IR	49.57 ± 9.88	47/38.5	25.8	185.9 ± 49.2	290.7 ± 63.3	8 ± 0.2

*NR* Not reported, *HRQL* Health-related quality of life, *T2DM* Type 2 diabetes mellitus

### Effect sizes

The effect sizes in the present study were event rates (ER). The rates for particular GI complications linked to metformin treatment and number of participants in each study has been presented in Supplementary Table 1. We found that the incidence of abdominal pain, bloating, constipation, diarrhea, nausea and vomiting were 5.3, 6.2, 1.1, 6.9, 5.0 and 2.4 percentages respectively.

#### Abdominal pain

**Fig. 2:**
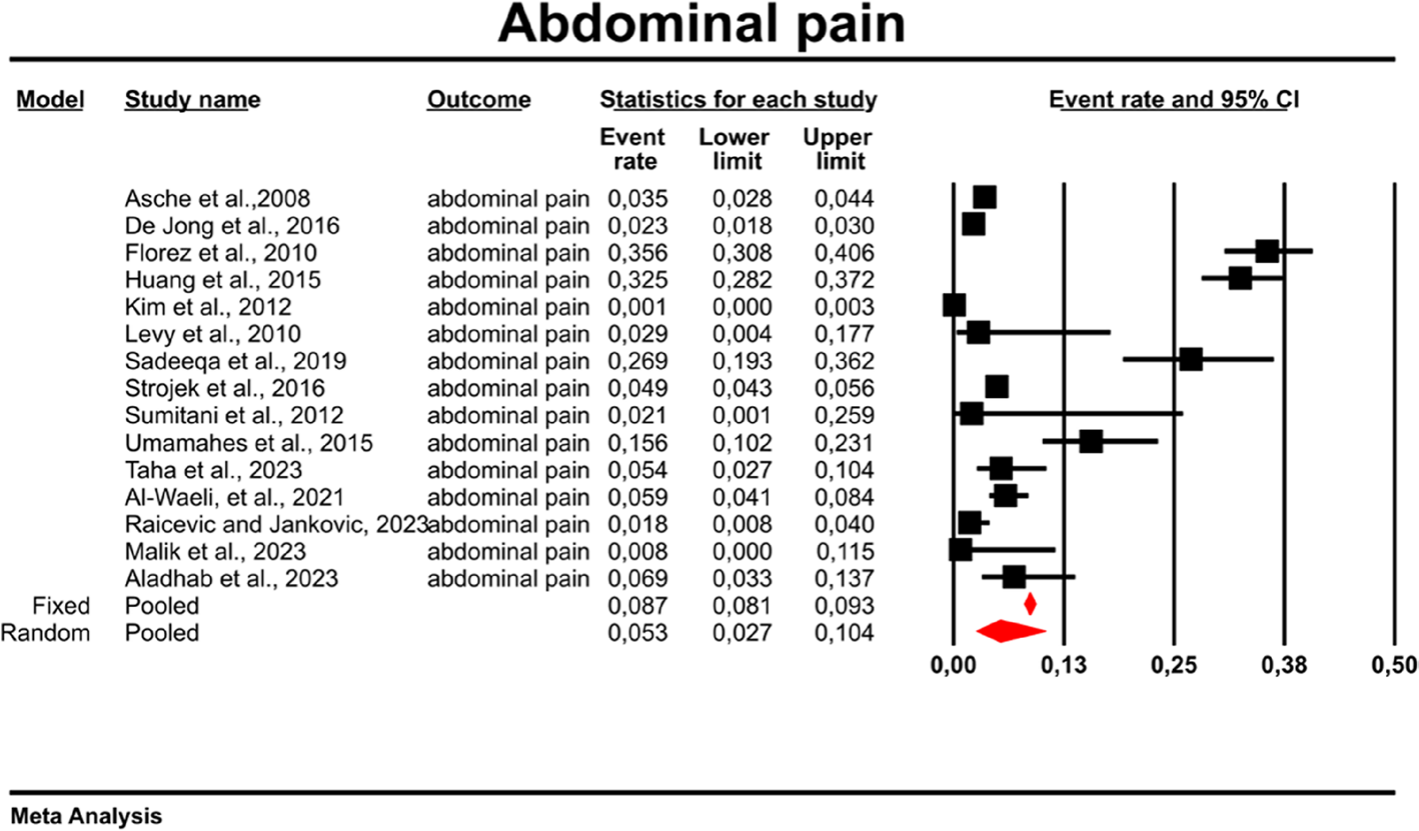
Event rates for abdominal pain in patients treated with metformin

Among the 15 studies that reported abdominal pain as AE, using a random-effects model, we found an abdominal pain rate of 0.053 with the prediction interval of 0.003 to 0.104 in patients treated with metformin (Fig. 2). There was however a high heterogeneity between studies as indicated by I^2^ measure: 98.442; *p* = 0.00; Q = 898.5; df = 14.

**Fig. 3:**
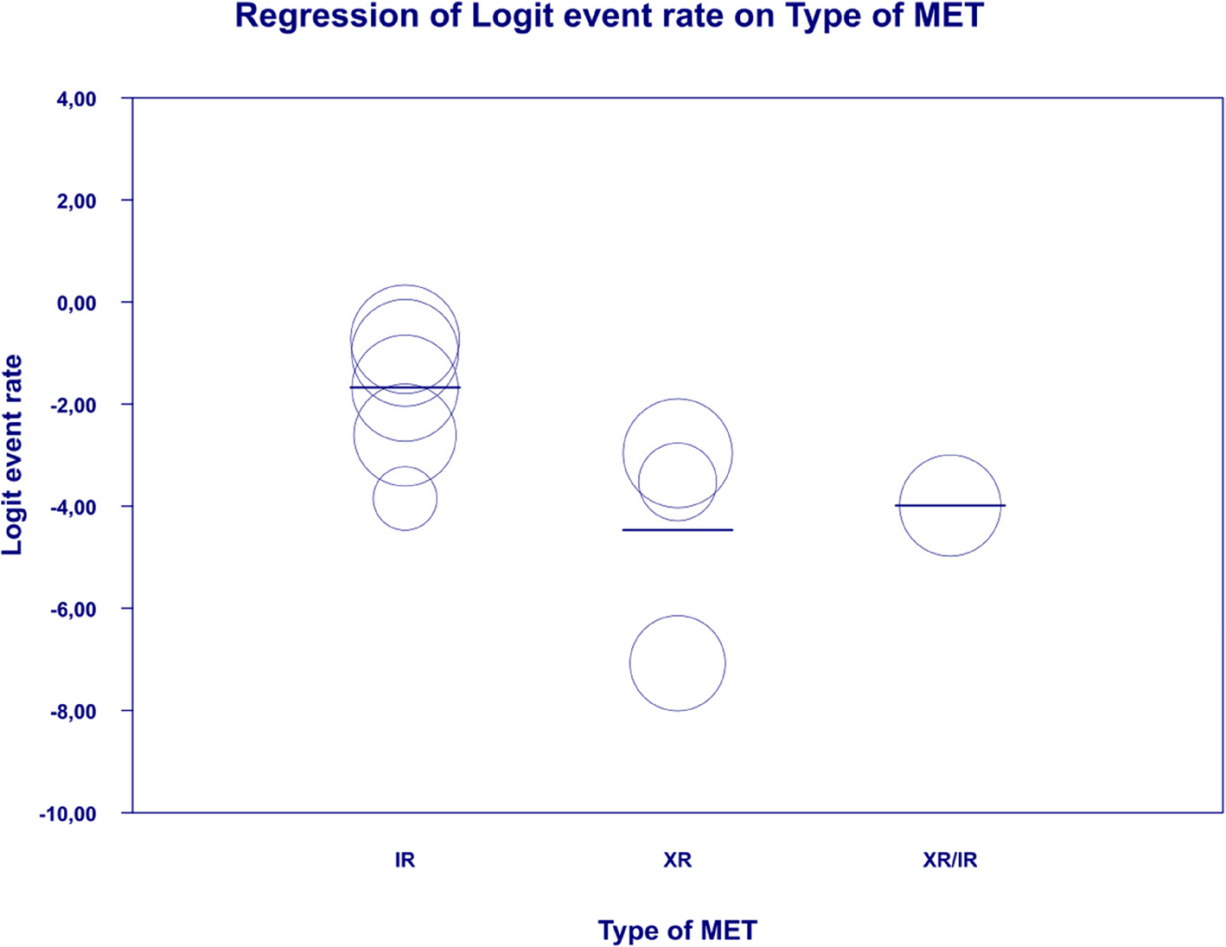
Regression for ER toward abdominal pain by type of metformin

**Fig. 4:**
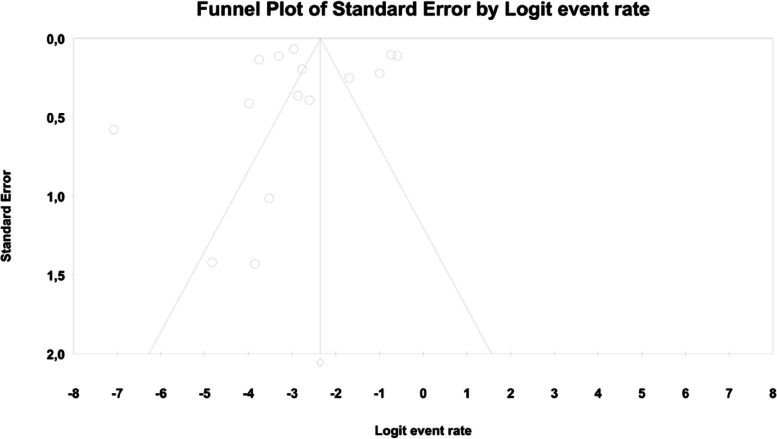
Funnel plot of standard error by logit event rate for abdominal pain

In meta-regression, the event rate for abdominal pain was significantly lower (Q = 12.1; df = 2; *p* = 0.0024) in XR and XR or IR compared to IR (XR coefficient -2.7985; SE: 0.85; 95% CI: -4.46 – -1.13; *p* = 0.001; vs. XR/IR coefficient: -2.3172; SE: 1.21; 95% CI: -4.70–0.067; *p* = 0.057) (Fig. 3). The dosage (coefficient 0.0004; SE: 0.0009; 95%CI: -0.0013–0.0022; *p* = 0.614); preexisting metformin treatment (coefficient -0.5752; SE: 1.1229; 95%CI: -2.7760–1.6256; *p* = 0.6085); average age (coefficient 0.0290; SE: 0.06; 95%CI: -0.0887–0.1466; *p* = 0.6296); sex (coefficient -0.0459; SE: 0.0353; 95%CI: -0.1152–0.0233; *p* = 0.1933); average BMI (coefficient -0.0010; SE: 0.1143; 95%CI: -0.2250–0.0231; *p* = 0.9933); average FBG (coefficient -0.0229; SE: 0.0207; 95%CI: -0.0634–0.0177; *p* = 0.2695); average PBG (coefficient 0.0077; SE: 0.0052; 95%CI: -0.0025–0.0178; *p* = 0.1386) and average HbA1c (coefficient 0.2448; SE: 0.4849; 95%CI: -0.7056–1.1952; *p* = 0.6137) showed no significant influence on the study-level effect sizes. After evaluating funnel plots, we determined through Egger’s test that there was no evidence of publication bias regarding the rate of abdominal pain (*p* = 0.69) (Fig. 4).

#### Bloating

**Fig. 5:**
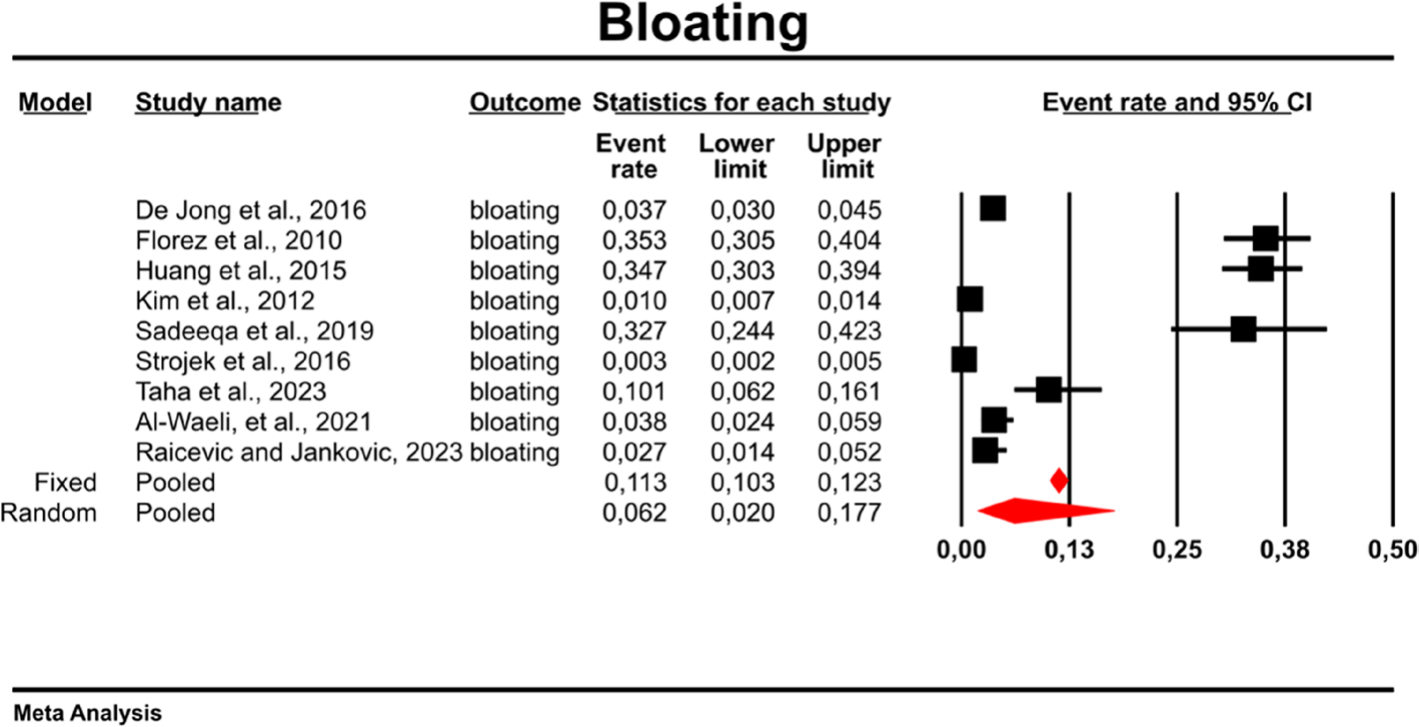
Event rates for bloating in patients treated with metformin

Using random-effect weights, we found that the overall rate for bloating was 0.062 (95%CI: 0.020–0.177) in patients with T2DM treated with metformin (Fig. 5). There was high heterogeneity across the studies (*I*^*2*^ = 99.207%, *p* = 0.00; Q = 1008.493; df = 8).

**Fig. 6:**
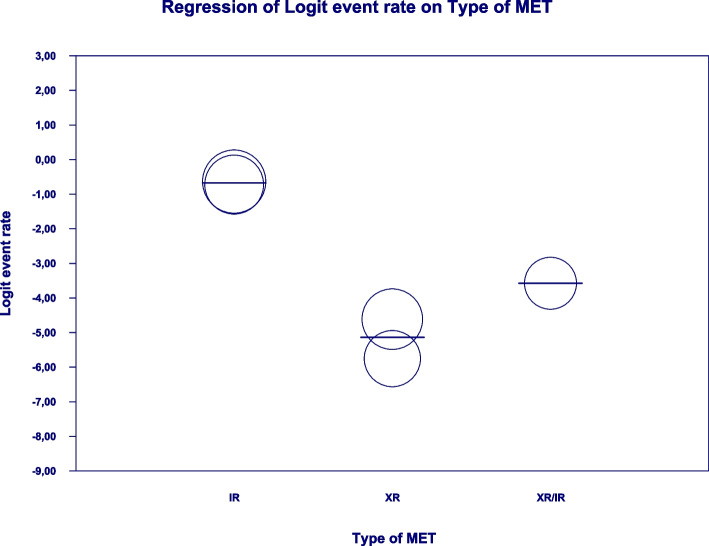
Regression for ER toward bloating by type of metformin

**Fig. 7:**
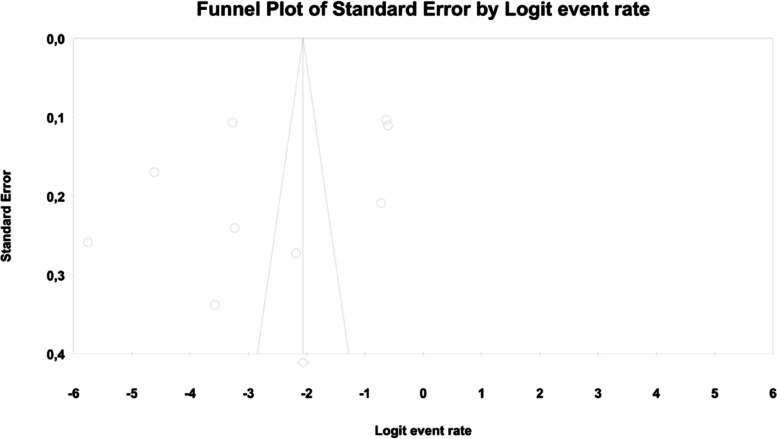
Funnel Plot of Standard Error by Logit event rate for bloating

In the case of meta-regression, the following covariates did not influence the effect size: dosage (coefficient -0.0008; SE: 0.0019; 95%CI: -0.0045–0.0028; *p* = 0.6495); pre-existence of MET treatment (coefficient 0.6976; SE: 1.6621; 95%CI: -2.5601–3.9552; *p* = 0.6747); average age (coefficient 0.0885; SE: 0.0997; 95%CI: -0.1069–0.2838; *p* = 0.3746); sex (coefficient -0.0690; SE: 0.0866; 95%CI: -0.2388–0.1009; *p* = 0.4261); average BMI (coefficient -0.1040; SE: 0.2902; 95%CI: -0.6729–0.4648; *p* = 0.7200); average FBG (coefficient -0.0026; SE: 0.0346; 95%CI: -0.0704–0.0652; *p* = 0.9405); average HbA1c (coefficient 0.9653; SE: 1.0641; 95%CI: -1.1203–3.0509; *p* = 0.3644). In contrast, the risk of bloating was elevated in persons receiving IR metformin when compared to XR drug (Q = 84; df = 2; *p* = 0.0000) (XR coefficient -4.4644; SE: 0.4896; 95% CI: -5.4240 – -3.5048; *p* = 0.0000) (Fig. 6). Due to the insufficient number of studies reporting on PBG, it was not incorporated into the meta-regression. Finally, we inspected funnel plots to find that Egger’s test did not suggest a publication bias regarding the ER of bloating (*p* = 0.224) (Fig. 7).

#### Constipation

**Fig. 8:**
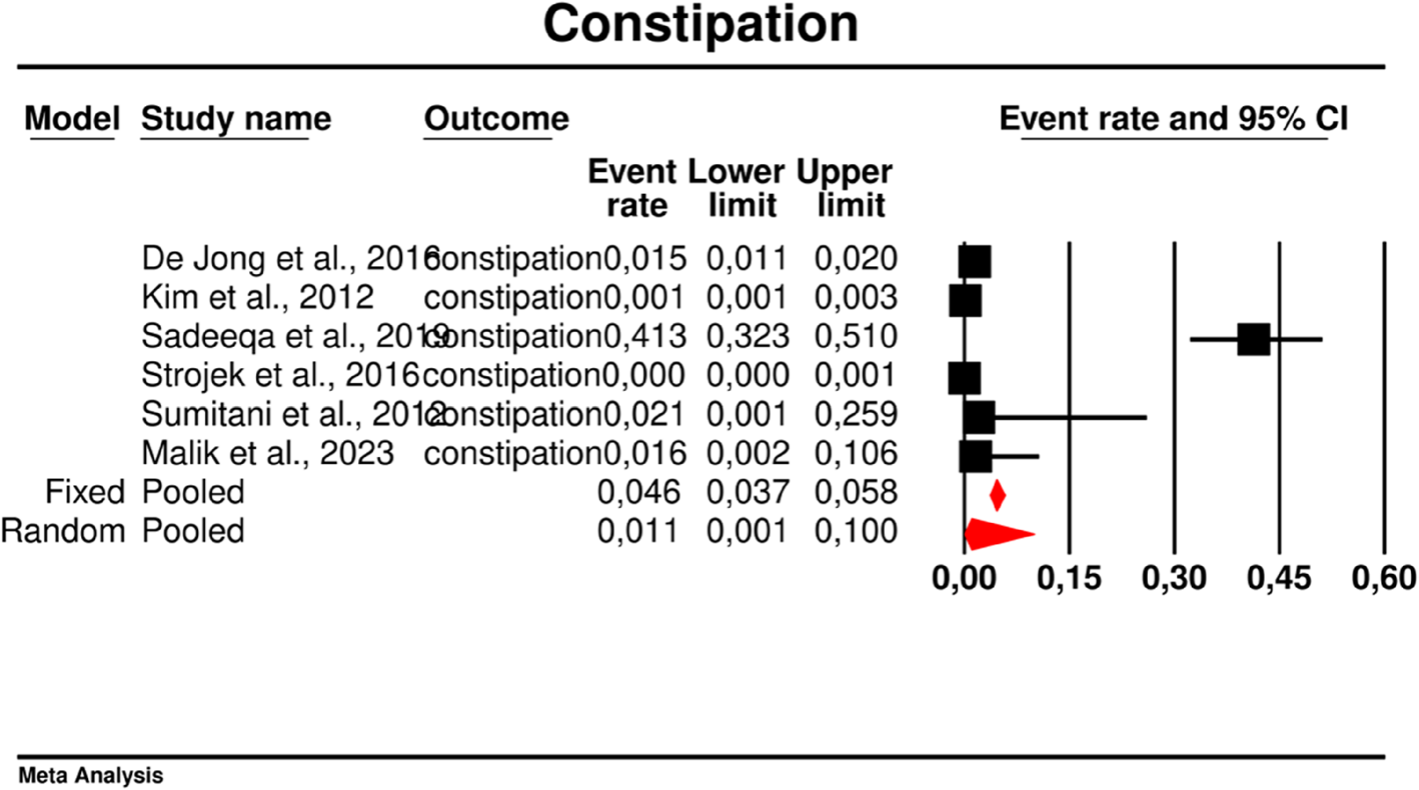
Event rates for constipation in patients treated with metformin

In the present study, the overall rate of constipation was 0.011 (95%CI: 0.001–0.100) (Fig. 8). There was high heterogeneity across the studies (*I*^*2*^ = 98.457%, *p* = 0.00; Q = 323,988; df = 5).

**Fig. 9:**
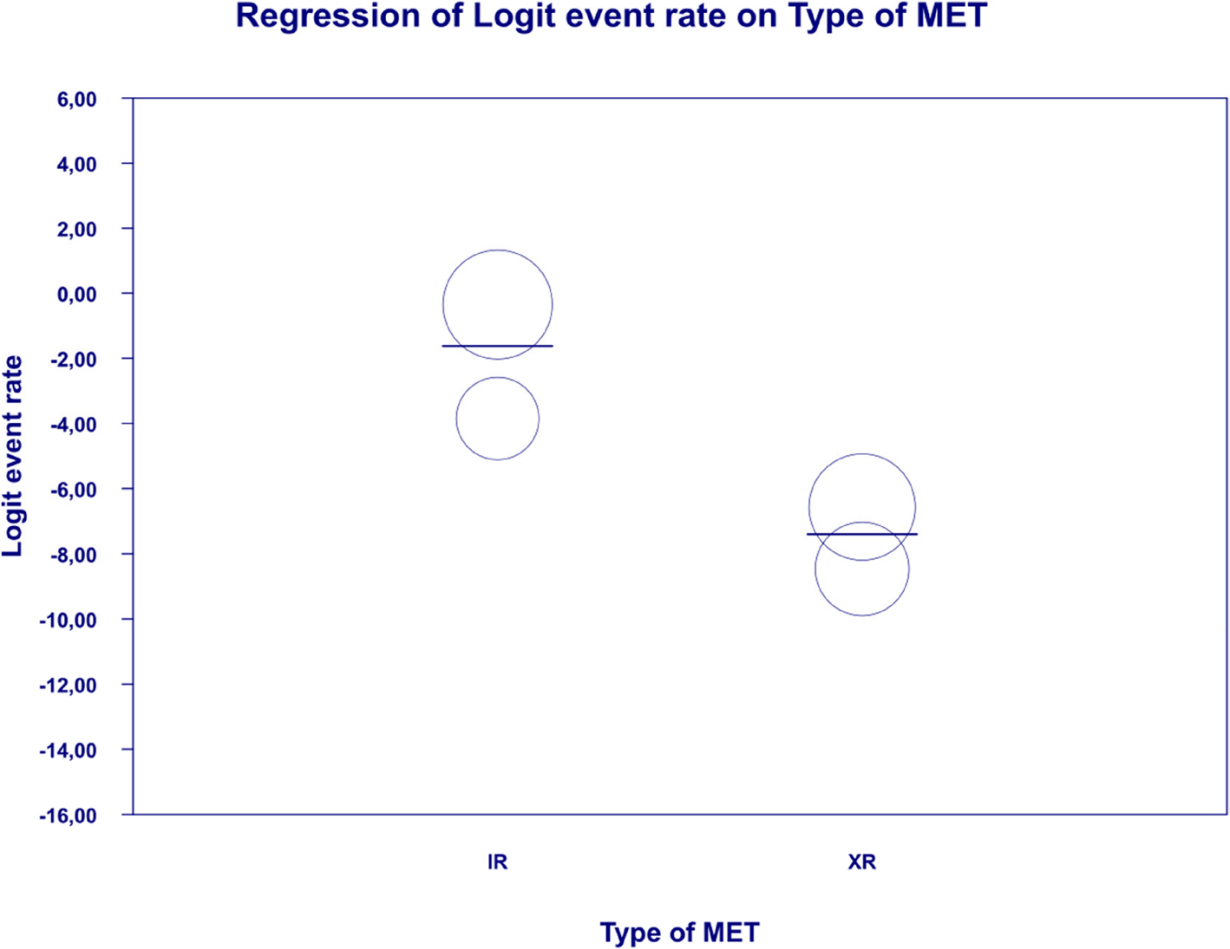
Regression for ER toward constipation by type of metformin

**Fig. 10:**
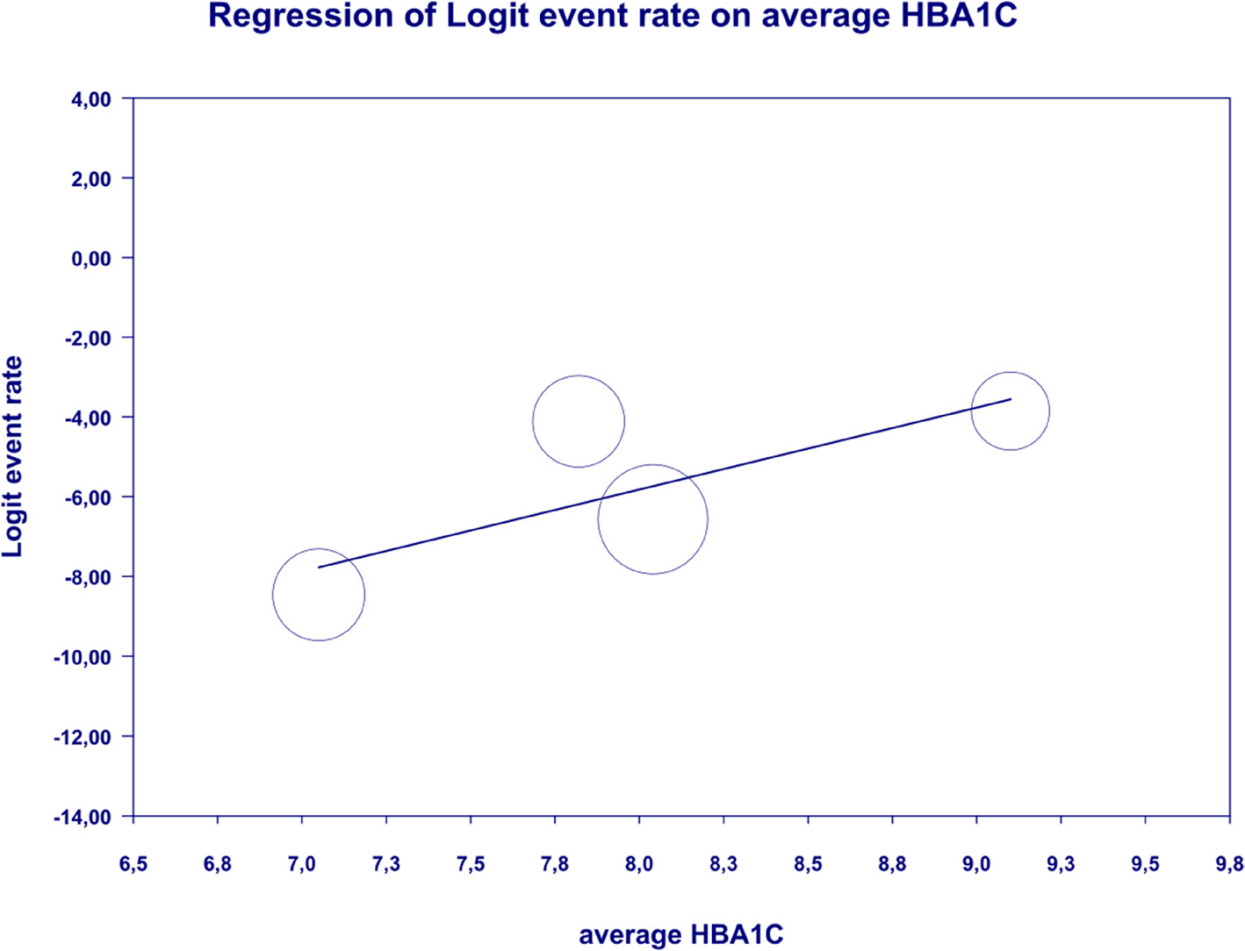
Regression for ER toward constipation by average HbA1c

**Fig. 11:**
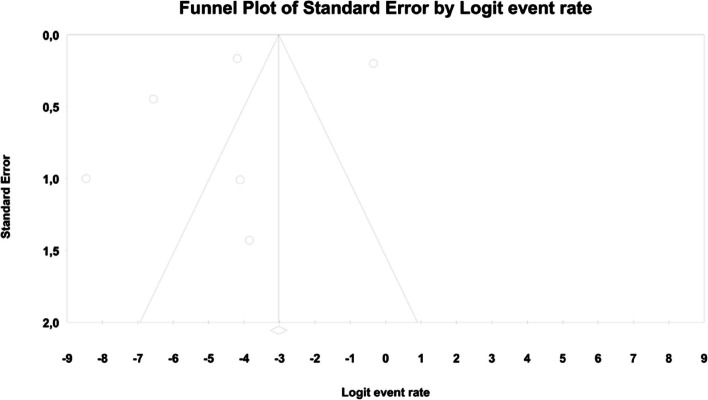
Funnel Plot of Standard Error by Logit event rate for constipation

In meta-regression, metformin XR was associated with a reduced rate of constipation risk (coefficient -5.7752; SE: 1.8110; 95%CI: -9.3247– -2.2257; *p* = 0.0014) (Fig. 9). There was a positive association between increases in HbA1c and the prevalence of constipation (coefficient 2.0582; SE: 1.2298; 95%CI: -0.3521–4.4685; *p* = 0.0942) (Fig. 10). The other covariates did not influence the effect size in the case of constipation: dosage (coefficient -0.0048; SE: 0.0046; 95%CI: -0.0139–0.0042; *p* = 0.2930); pre-existence of MET (coefficient -0.7759; SE: 2.5692; 95%CI: -5.8115–4.2597; *p* = 0.7627); average age (coefficient -0.4057; SE: 0.4554; 95%CI: -1.2983–0.4868; *p* = 0.3730); sex (coefficient -0.0244; SE: 0.0780; 95%CI: -0.1772–0.1284; *p* = 0.7546); BMI (coefficient -0.2422; SE: 0.5974; 95%CI: -1.4131–0.9286; *p* = 0.6851); average FBG (coefficient 0.0147; SE: 0.0410; 95%CI: -0.0657–0.0952; *p* = 0.7198). Due to the insufficient number of studies reporting on PBG, it was not incorporated into the meta-regression. Egger’s test did not suggest publication bias regarding the rate of nausea (*p* = 0.54) (Fig. 11).

#### Diarrhea

**Fig. 12:**
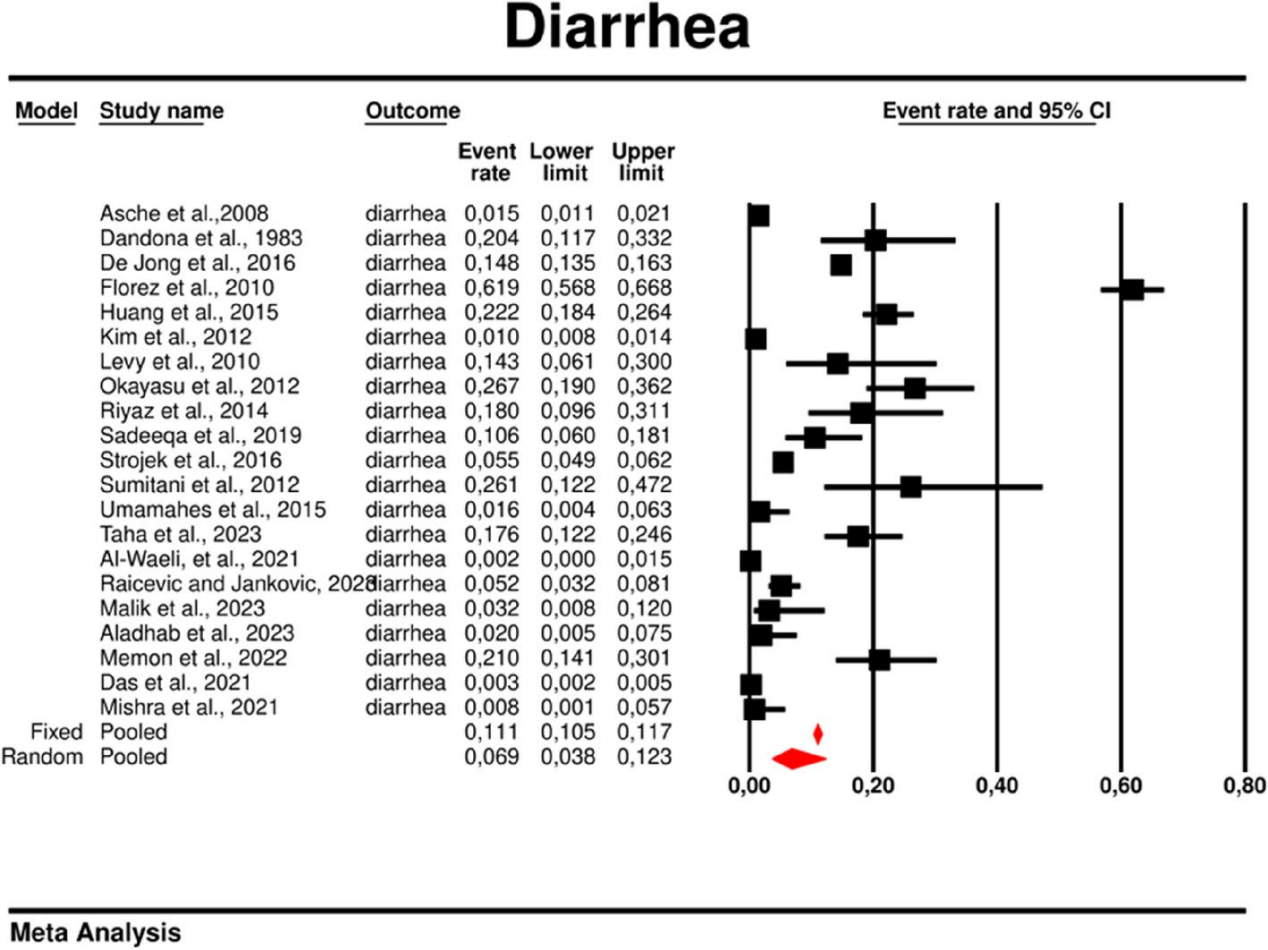
Event rates for diarrhea in patients treated with metformin

We reported diarrhea in all 21 studies included in this meta-analysis. The overall rate of diarrhea was significantly elevated and reached 0.069 (95%CI: 0.038–0.123) (Fig. 12). There was substantial heterogeneity across the studies (*I*^*2*^ = measure: 98.670; *p* = 0.00; Q = 1503.352; df = 20)

**Fig. 13:**
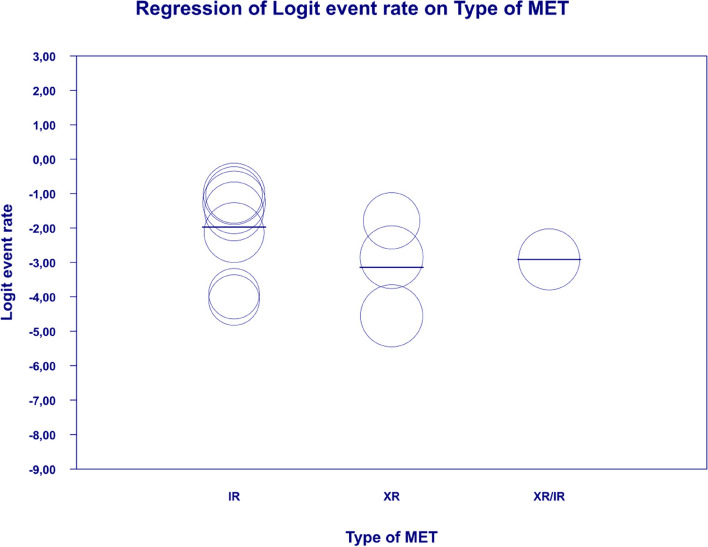
Regression for ER toward diarrhea by type of metformin

**Fig. 14:**
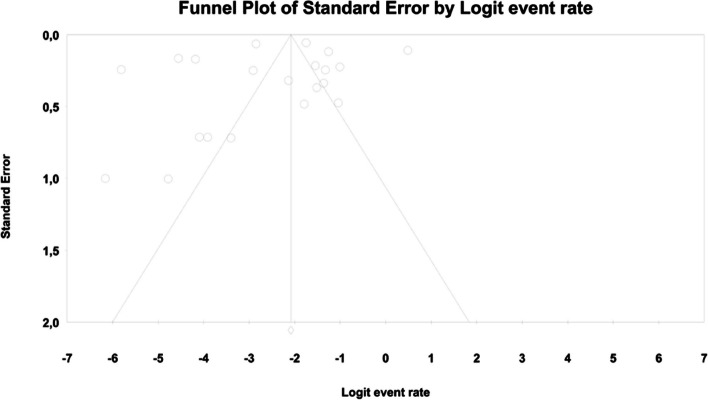
Funnel Plot of Standard Error by Logit event rate for diarrhea

. The following covariates were not significantly linked to the effect size: dosage (coefficient -0.0004; SE: 0.0007; 95%CI: -0.0017–0.0009; *p* = 0.5191); pre-existence of MET (coefficient 0.4348; SE: 0.8818; 95%CI: -1.2935–2.1631; *p* = 0.6220); average age (coefficient 0.0485; SE: 0.0553; 95%CI: -0.0599–0.1568; *p* = 0.3808); sex (coefficient 0.0098; SE:0.321; 95%CI: -0.0531–0.0727; *p* = 0.7593); average BMI (coefficient -0.0748; SE: 0.1410; 95%CI: -0.3511–0.2015; *p* = 0.5958); average FBG (coefficient 0.0077; SE: 0.0141; 95%CI: -0.0199–0.0352; *p* = 0.5853); average PBG (coefficient 0.0082; SE: 0.0079; 95%CI: -0.0073–0.0237; *p* = 0.2980); average HbA1c (coefficient 0.3433; SE: 0.2528; 95%CI: -0.1522– 0.8387; *p* = 0.1745). In persons who received the XR MET treatment formulation we found fewer GI events compared to patients treated with IR MET formulation (coefficient -1.1715; SE: 0.7020; 95%CI: -2.5473– 0.2043; *p* = 0.0951) (Fig. 13). Egger’s test did not suggest a publication bias regarding the ER of diarrhea (*p* = 0.497) (Fig. 14).

#### Nausea

**Fig. 15:**
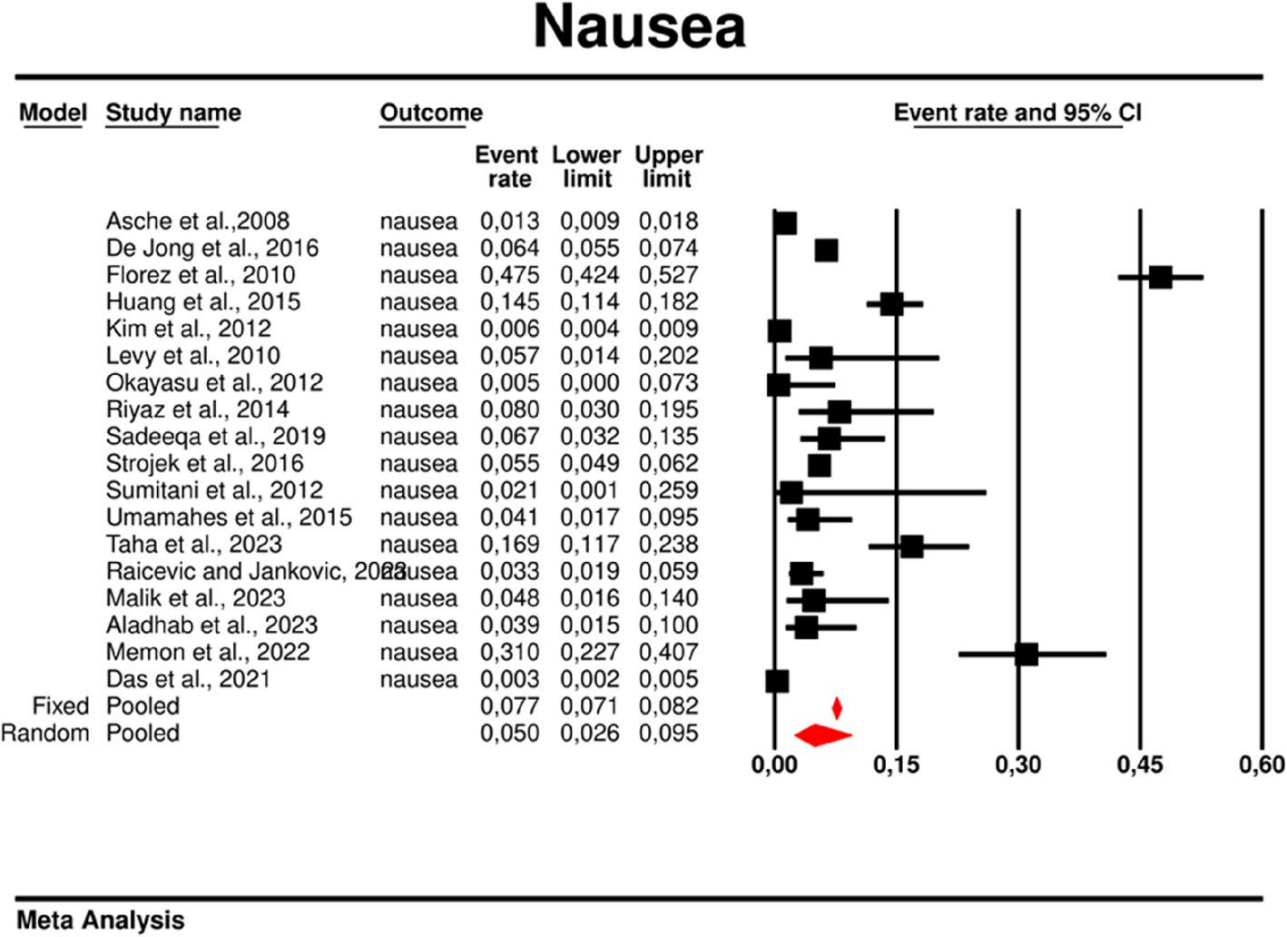
Event rates for nausea in patients treated with metformin

Using random-effects weights, we found that the overall rate of nausea was 0.05 (95%CI: 0.026–0.095) (Fig. 15). There was high heterogeneity across the studies (*I*^*2*^ = 98.468%, *p* = 0.00; Q = 1109.713; df = 17).

**Fig. 16:**
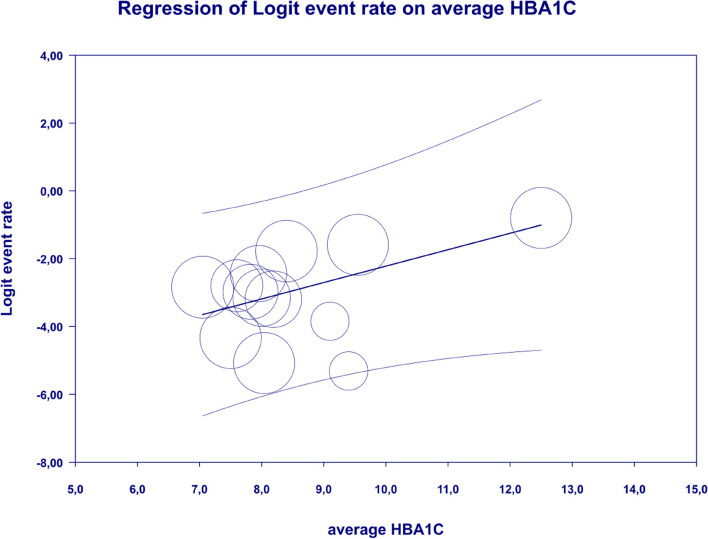
Regression for ER toward diarrhea by type of metformin

**Fig. 17:**
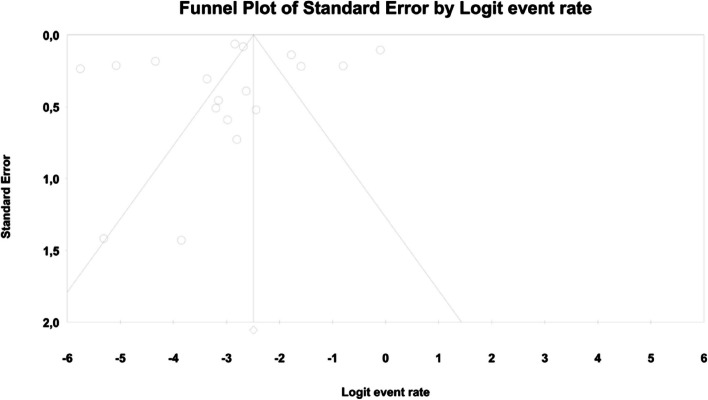
Funnel Plot of Standard Error by Logit event rate for nausea

In the case of meta-regression, the following covariates did not influence the effect size: dosage (coefficient -0.0000; SE: 0.0007; 95%CI: -0.0013–0.0013; *p* = 0.9547); pre-existence of MET (coefficient 0.3691; SE: 1.0456; 95%CI: -1.6801– 2.4184; *p* = 0.7241); type of metformin (XR coefficient -0.6984; SE: 0.9218; 95%CI: -2.5051–1.1083; *p* = 0.4486); average age (coefficient 0.0097; SE: 0.0552; 95%CI: -0.0985–0.1179; *p* = 0.8608); sex (coefficient -0.0447; SE:0.0332; 95%CI: -0.1097–0.0204; *p* = 0.1785); average BMI (coefficient 0.1600; SE: 0.1354; 95%CI: -0.1053–0.4254; *p* = 0.2372); average FBG (coefficient 0.0135; SE: 0.0138; 95%CI: -0.0136–0.0406; *p* = 0.3287); average PBG (coefficient 0.0110; SE: 0.0073; 95%CI: -0.0033–0.0253; *p* = 0.1308). In contrast, the risk of nausea was elevated in persons with higher level of HbA1c parameter (coefficient 0.4846; SE: 0.2398; 95%CI: 0.0146– 0.9547; *p* = 0.0433) (Fig. 16). Through Egger’s test we determined that there was no evidence of publication bias regarding the rate of nausea risk (*p* = 0.53) (Fig. 17).

#### Vomiting

**Fig. 18:**
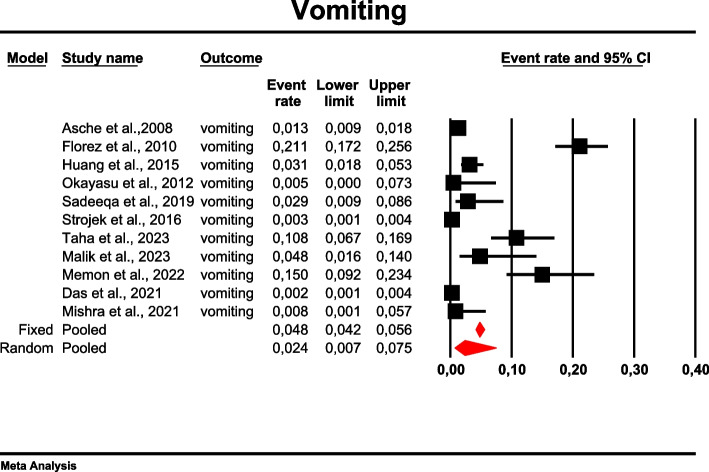
Event rates for vomiting in patients treated with metformin

The overall risk for vomiting among patients with TD2M treated with metformin reached 0.024 (95%CI: 0.007–0.075) (Fig. 18). There was high heterogeneity across the studies (*I*^*2*^ = 97.981%, *p* = 0.00; Q = 495.329; df = 10).

**Fig. 19:**
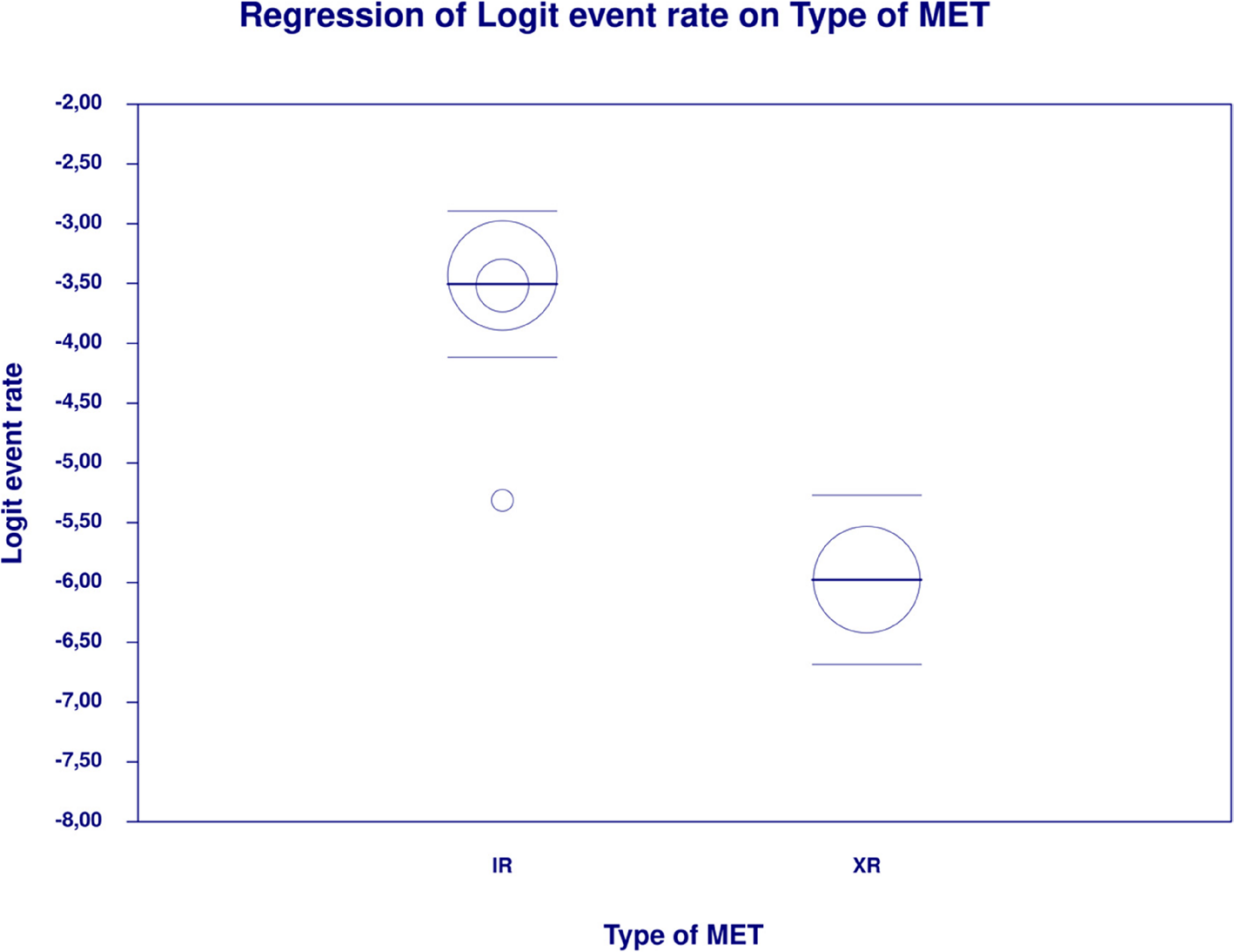
Regression for ER toward diarrhea by type of metformin

**Fig. 20:**
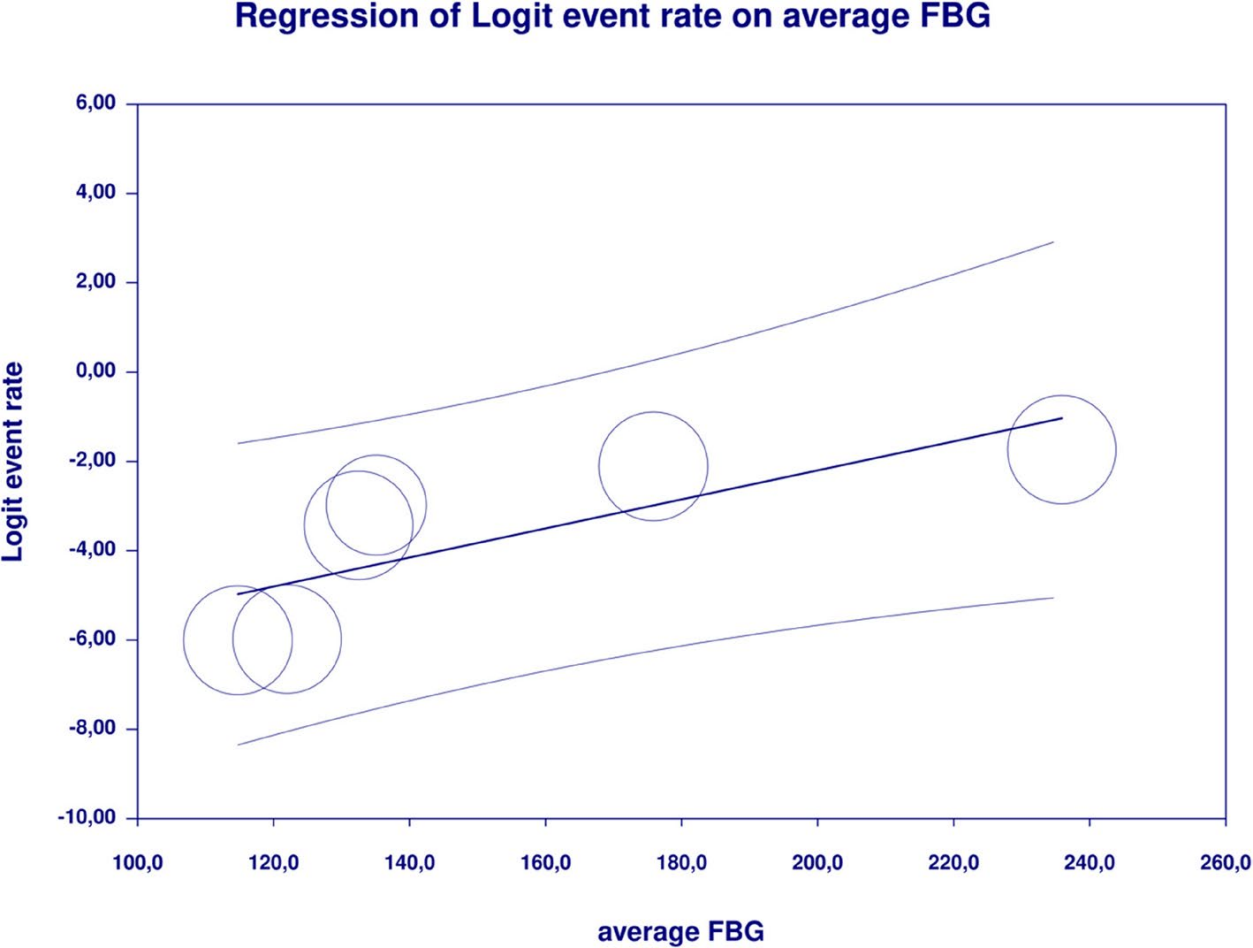
Regression for ER toward diarrhea by average FBG

**Fig. 21:**
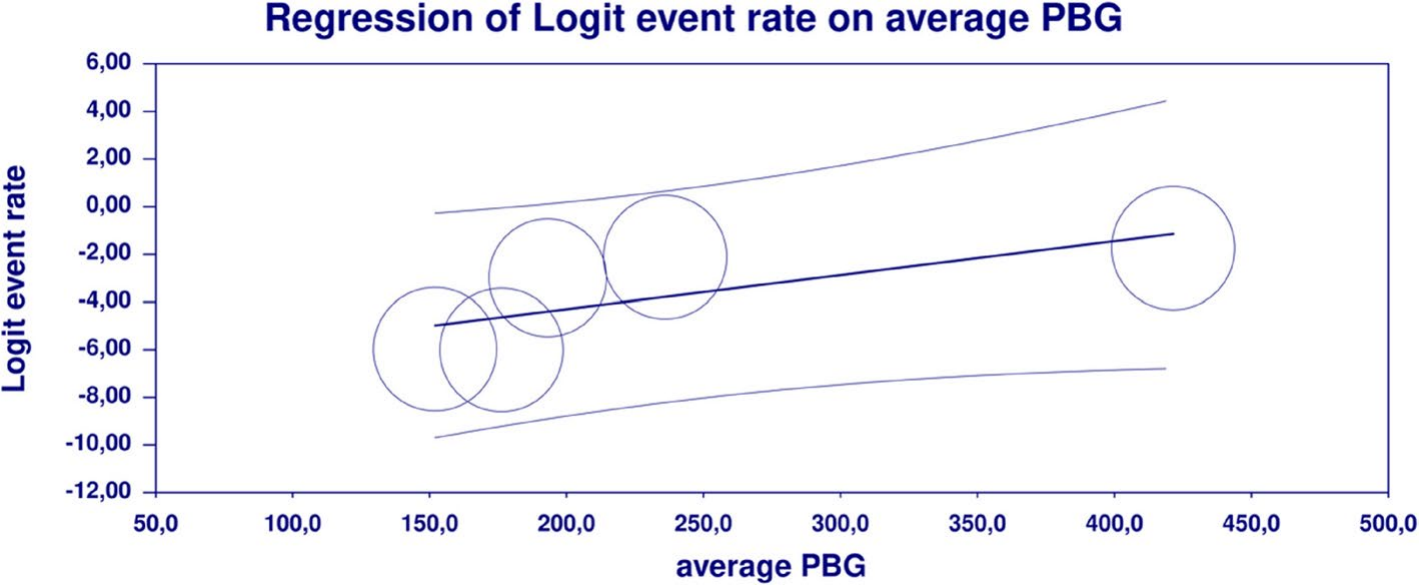
Regression for ER toward diarrhea by average PBG

**Fig. 22:**
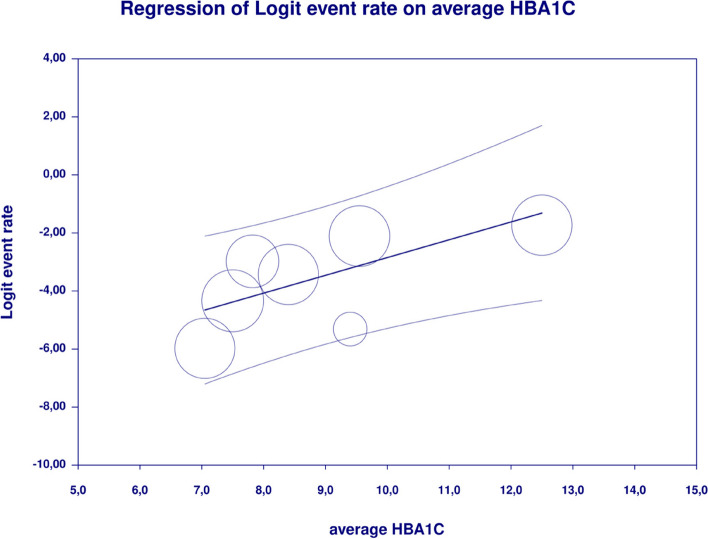
Regression for ER toward diarrhea by average HbA1c

**Fig. 23:**
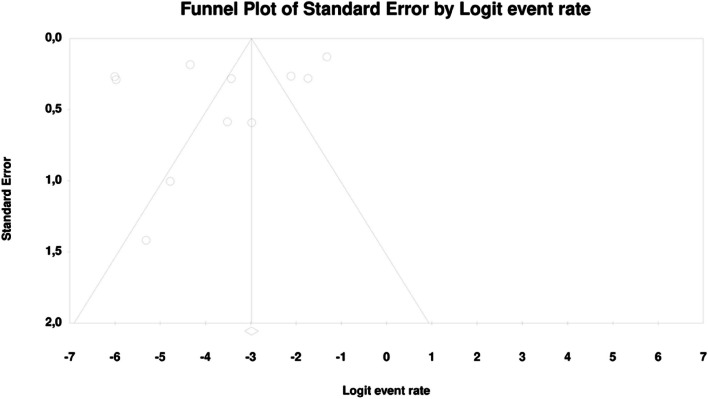
Funnel Plot of Standard Error by Logit event rate for vomiting

In meta-regression, metformin XR was associated with a reduced rate of vomiting (XR coefficient -2.4702; SE: 0.3821; 95%CI: -3.2192– -1.7213; *p* = 0.0000) (Fig. 19). Similarly, the risk of vomiting was increased for elevated levels of the following variables: average FBG (coefficient 0.0325; SE: 0.120; 95%CI: 0.0090–0.0560; *p* = 0.0067) (Fig. 20), average PBG (coefficient 0.0142; SE: 0.0077; 95%CI: -0.009–0.0294; *p* = 0.0653) (Fig. 21), average HbA1c (coefficient 0.6131; SE: 0.2089; 95%CI: 0.2037–1.0224; *p* = 0.0033) (Fig. 22). Whereas dosage (coefficient -0.0005; SE: 0.0013; 95%CI: -0.0030–0.0020; *p* = 0.7042); pre-existence of MET (coefficient 1.8124; SE: 1.3375; 95%CI: -0.8091–4.4338; *p* = 0.1754); average age (coefficient -0.0126; SE: 0.0957; 95%CI: -0.2001– 0.1749; *p* = 0.8949); sex (coefficient -0.0816; SE:0.0625; 95%CI: -0.2042–0.0409; *p* = 0.1918); average BMI (coefficient 0.1244; SE: 0.2661; 95%CI: -0.3971–0.6460; *p* = 0.6400) did not associate with the effect size. Egger’s test did not suggest publication bias regarding the RR of nausea (*p* = 0.3) (Fig. 23).

### The risk of *bias* of included studies

By means of NOS tool, we estimated that the mean number of stars was 6.1 ± 1.38 (median 7). The highest score, i.e. 8 was demonstrated for 2 studies [22, 41] whilst only 1 study presented the lowest score, i.e. 3 [34]. The details are presented in Supplementary Table 2.

## Discussion

In this systematic review and meta-analysis with meta-regression of observational studies of metformin treatment in patients with T2DM, our key findings are as follows: (i) we have estimated the occurrence of GI AE in the real world data based on observational studies, (ii) diarrhea is the most common GI AE of metformin treatment and described in all analyzed studies (iii) the use of metformin XR formulation is associated with lower incidence of abdominal pain, bloating, constipation, diarrhea, and vomiting compared to metformin IR, and (iv) the prevalence of GI AE was not associated with the dose size of metformin nor prior metformin treatment therapy, mean age, gender (as % of males) and mean BMI. These results demonstrate similar prevalence of GI AE to a recently published phase 2 RCT in patients with prediabetes and concomitant HIV, where a higher prevalence would theoretically have been hypothesized [42].

Based on RCTs, the different GI-related AE may occur in patients with T2DM treated with metformin which limits its use [43]. In our recent systematic review, meta-analysis and meta-regression of RCT, we found that metformin use was associated with a higher risk of abdominal pain, diarrhea and nausea compared to other glucose-lowering drugs or placebo [17]. In the current study, we did not evaluate the side effects of metformin in comparison to other glucose-lowering drugs. Instead, we report the event rate of AE from real world observational data.

Diarrhea emerged as the most frequently observed AE, affecting 6.9% of the population treated with metformin. This observation aligns with the results of our prior meta-analysis of RCT. Further, in both meta-analyses the frequency of other GI AE followed a consistent descending order: bloating, abdominal pain, nausea, vomiting, and constipation. This pattern suggests a robust trend in the manifestation of these GI AE in patients treated with metformin [17]. There are multiple potential systemic effects of metformin that contribute to this side effect. By its structural relation to selective agonists of the serotonin 5-HT3 receptor, induces 5-HT3 receptor independent release of 5-HT from human duodenal mucosa, what may cause GI symptoms, including diarrhea [44]. Additionally, metformin treatment leads to disturbances in the entero-hepatic circulation of bile salts and outcome osmotic diarrhea, through reducing ileal bile salt reabsorption and leading to elevated colonic bile salt concentrations [45]. Moreover, microbiome alterations caused by metformin can be responsible for its different side-effects [46]. Increased osmotic burden in the colon is a possible explanation for watery stool formation in patients on metformin particularly at the treatment beginning [45].

The results of the presented meta-analysis are characterized by high heterogeneity, which means that the findings from different studies included in the meta-analysis vary significantly. The observed variation may stem from differences in study designs, characteristics of the study population, research methodologies and contextual factors. For example, in Florez et al. research, where the aim of the study was to determine metformin-associated GI symptoms in treatment-naïve patients with type 2 diabetes, the most commonly reported GI symptom was diarrhea which occurred in 62.1% of patients [31]. Huang et al. assessed whether Helicobacter pylori infection could influence metformin tolerance in patients with type 2 diabetes mellitus by comparing two groups of patients those with and without H. pylori infection [21]. In this study, the most commonly reported symptom in patients without infection was bloating (34.7%), whereas diarrhea appeared in 22.2% of subjects [21]. Sadeeqa et al. concluded that GI intolerance increased with the higher dose of metformin and the most commonly occurring adverse effect was constipation, while diarrhea only in 6.58% of patients [38]. It is worth considering the reason for this several-fold increase in the rate of diarrhea in patients T2DM patients treated with metformin from Florez et al. study [31].

To explain these notable discrepancies in frequency but also type of GI AE, we investigated the effect of metformin dose size, prior metformin treatment and formulation of metformin on its tolerance using meta regression technique.

### Metformin dose size

According to ADA and EASD consensus report, GI symptoms are dose dependent, and may improve with dose reduction, therefore the dosage should be increased as tolerated to a target optimal dose [4]. Although the incidence of digestive disturbances has been reported to be dose-related [40], there was no association between the incidence of AE and metformin dosage in the present analysis. Similarly, in other studies there was no relationship between dosage and incidence of GI AE [44].

It is worth emphasizing that the relationship between metformin dose and GI AE may be influenced by other factors, such as individual genetic predisposition to metformin intolerance, mainly OCT1 polymorphisms leading to intolerance through increasing metformin concentration in the intestine [47]. Moreover, Dujic et al. demonstrated that the concomitant use of OCT1-inhibiting medications like: citalopram, proton pump inhibitors, verapamil, doxazosin, and codeine was significantly associated with metformin intolerance, whereby verapamil increased the odds of intolerance sevenfold [48].

### Prior metformin treatment

Initiation of metformin treatment in our study was not associated with an increased prevalence of any GI AE. Metformin-associated diarrhea typically appears during the commencement of treatment and subsides after cessation of therapy [28]. Yuxin et al. reported that most discontinuations of treatment due to metformin intolerance occurred in the first third of the length of the trial [49]. According to ADA and EASD consensus report, GI AE may improve over time [4].

### Formulation of metformin

In terms of glucose lowering potency, the efficacy of the two formulations: XR and IR release is similar, however XR formulation is associated with fewer gastrointestinal side-effects [50, 51]. The UK NICE guidelines recommend the use of metformin XR in patients intolerant to metformin IR [52]. Observed improvements in GI AE with the XR formulation may be due to the tablet design, which releases metformin slowly and subsequently decreases GI exposure to the drug [53]. In the present meta-analysis, we used meta-regression to examine the effect of metformin formulation on effect estimates and we found positive correlation between XR formulation and fewer GI side effects like abdominal pain, bloating, constipation, diarrhea, and vomiting. In most studies, the type of metformin was an important moderator, although it is important to mention that this result should be treated with caution, as some researchers reported a mean metformin dose when others a maximum dose. There are many studies, among them our recent meta-analysis [17], which indicate that XR metformin may be a better option for patients who have GI intolerance using the IR formulation, while still achieving glycemic control [54, 55]. Another meta-analysis did not confirm reduced GI AE with metformin XR, however, it led to improvement in compliance [18].

### Age, gender, mean BMI

In this study, there was no association between age, gender (as % of males) and BMI on metformin tolerability. Clinical evidence supports the efficacy of metformin for weight loss in patients with diabetes mellitus and overweight or obesity [56]. Of note, patients with obesity, higher FBG and younger age were more likely to respond to metformin in the study by Aroda et al. [57], although the effect of these factors on metformin tolerability and GI AE is not mentioned. Gender differences were observed in the treatment patterns of patients with T2D after starting metformin. Women switched treatment more often than men and were more likely to switch to another non-insulin glucose lowering gent after starting metformin, whereas the time to treatment intensification was shorter in men. However, the study highlights potential differences in diabetes management between men and women, the causes and consequences of which should be further investigated [58].

### FBG, PBG, HbA1c

This study showed that the risk of GI AE such as constipation (*p* = 0.0942), nausea (*p* = 0.0433) and diarrhea (*p* = 0.0033) increased with higher HbA1c levels. Similarly high FBG (*p* = 0.0067) and PBG (*p* = 0.0653) levels leads to increased likelihood of vomiting. In the studies included in the meta-analysis, HbA1c, FBG and PBG was measured at baseline, during and after metformin treatment, which may have influenced the results. In uncontrolled diabetes, with high FBG and PBG levels, rising HbA1c levels prompt the initiation of glucose lowering treatment, typically metformin, which may be associated with an increased risk of adverse events at high HbAc. Metformin therapy may reduces HbA1c levels in a clinically meaningful way by an average of about 1–2 percentage points [59].

## Strengths and limitations

To the best of our knowledge, this analysis provides the first systematic review with meta-analysis and meta-regression of observational studies regarding the risk of GI AE in patients with T2DM treated with metformin. In the evaluation of GI AE associated with metformin use, our study leverages observational data to complement previous meta-analysis conducted in RCTs [17]. Observational studies offer an expanded purview of real-world clinical outcomes, capturing a diverse range of patients often excluded from RCTs due to stringent inclusion and exclusion criteria [60]. This is particularly relevant given the routine practice of "run-in" periods in RCTs, which effectively screen out participants who do not initially tolerate the drug, thus potentially underrepresenting the true incidence of GI AE in the general population of patients with T2DM [61]. By focusing on observational data, our analysis aims to provide a more comprehensive and generalizable assessment of the gastrointestinal risks associated with metformin use.

The substantial variance in point estimates, as reflected in the confidence intervals, can be attributed to a limited number of studies and large variability of the results of included studies, possibly due to potential confounders, such as the concomitant use of metformin with other glucose-lowering agents.

## Conclusion

The AE such as diarrhea, bloating, abdominal pain, nausea, vomiting and constipation in T2DM patients treated with metformin are common, with diarrhea being the most prevalent. The use of metformin XR formulation is associated with lower risk of GI AE compared to IR formulation. However, the risk of GI AE is not associated with the dose size of metformin nor prior metformin treatment.

## Supplementary Information

Supplementary Material 1.

## Data Availability

Data is provided within the manuscript or supplementary information files. Further inquiries can be directed to the corresponding author.
