# Supplementary material for: Gastrointestinal adverse events of metformin treatment in patients with type 2 diabetes mellitus: a systematic review and meta-analysis with meta-regression of observational studies
Source: BMC Endocr Disord. 2024 Sep 30;24:206. doi: 10.1186/s12902-024-01727-w (PMC11440709; doi:10.1186/s12902-024-01727-w)
Supplement: Supplementary file 1 — Supplementary Material 1. [file 12902_2024_1727_MOESM1_ESM.docx]

Supplementary Material

# Supplementary Data

**Search Strategy and Inclusion/Exclusion Criteria**

There were two independent authors (KI and MH) who searched five certain bibliographic databases (PUB MED/Cinahl/Web of Science/Scopus/Embase) from database inception until 08/11/2020 for studies evaluating GI AEs of metformin treatment in patients with type 2 diabetes. Our target was data provided in observational trials only.

The following search terms were used:

PUB MED/Cinahl/Web of Science/Scopus/

(diabetes mellitus OR diabetes OR diabetes mellitus OR diabetic OR insulin resistance OR insulin resistance OR resistance, insuline) AND (metformin OR 1, 1 dimethylbiguanide OR dimethyldiguanide OR dimethylbiguanide OR metformin OR metformina OR metformine OR methformin OR metiguanide OR metphormin OR n` dimethylguanylguanide OR n` dimethylguanylguanidine OR n`, n` dimethyldiguanide OR n, n dimethyl biguanidine OR n, n dimethylbiguanide OR n, n dimethyldiguanide OR n, n dimethylbiguanidine OR n, n dimethylbiguanide retard OR n, n dimethylguanylguanidine) AND (abdominal pain OR abdomen pain OR abdominal pain OR pain, abdominal OR nausea and vomiting OR nausea and emesis OR nausea and vomiting OR nausea emesis OR bloating OR abdominal bloating OR bloating OR diarrhea OR diarrhea OR diarrhea, toxic OR diarrhoea OR diarrhoea, toxic OR postoperative diarrhea OR postoperative diarrhoea OR scour OR toxic diarrhea OR toxic diarrhoea) NOT (review OR meta analysis OR systematic review)

EMBASE

('diabetes mellitus' OR 'diabetes' OR 'diabetes mellitus' OR 'diabetic' OR 'insulin resistance' OR 'insulin resistance' OR 'resistance, insuline') AND ('metformin' OR '1, 1 dimethylbiguanide' OR 'dimethyldiguanide' OR 'dimethylbiguanide' OR 'metformin' OR 'metformina' OR 'metformine' OR 'methformin' OR 'metiguanide' OR 'metphormin' OR 'n` dimethylguanylguanide' OR 'n` dimethylguanylguanidine' OR 'n`, n` dimethyldiguanide' OR 'n, n dimethyl biguanidine' OR 'n, n dimethylbiguanide' OR 'n, n dimethyldiguanide' OR 'n, n dimethylbiguanidine' OR 'n, n dimethylbiguanide retard' OR 'n, n dimethylguanylguanidine') AND ('abdominal pain' OR 'abdomen pain' OR 'abdominal pain' OR 'pain, abdominal' OR 'nausea and vomiting' OR 'nausea and emesis' OR 'nausea and vomiting' OR 'nauseaemesis' OR 'bloating' OR 'abdominal bloating' OR 'bloating' OR 'diarrhea' OR 'diarrhea' OR 'diarrhea, toxic' OR 'diarrhoea' OR 'diarrhoea, toxic' OR 'postoperative diarrhea' OR 'postoperative diarrhoea' OR 'scour' OR 'toxic diarrhea' OR 'toxic diarrhoea') NOT ('review' OR 'meta analysis' OR 'systematic review')

ClinTrials: metformin | Completed Studies | Diabetes Mellitus (completed studies as a filter)

1. **Inclusion criteria:**

- observational studies with MET intervention in patients with type 2 diabetes mellitus
- populations containing >20 patients
- abstraction of data from metformin-treatment arms only (MET-treatment), however in the case of multiple arms data from all containing MET-treatment were abstracted
- available data on the incidence of any gastrointestinal adverse event following metformin administration, such as abdominal pain, diarrhea, bloating, flatulence, constipation, nausea and vomiting

1. **Exclusion criteria:**

- adverse events related to metformin treatment other than gastrointestinal
- another than English language of publication.

Table 1. GI complications in persons receiving metformin.

| **Reference** | **discontinuation due to adverse events** | | | **abdominal pain (n)** | | **diarrhea (n)** | | **vomiting (n)** | | **bloating (n)** | | **nausea (n)** | | **constipation (n)** | |
| --- | --- | --- | --- | --- | --- | --- | --- | --- | --- | --- | --- | --- | --- | --- | --- |
|  | **cases** | **n total** | **type of AE** | **cases** | **n total** | **cases** | **n total** | **cases** | **n total** | **cases** | **n total** | **cases** | **n total** | **cases** | **n total** |
| Aladhab et al., 2023 ^24^ | NR | NR | NR | 7 | 102 | 2 | 102 | NR | NR | NR | NR | 4 | 102 | NR | NR |
| Alibrahim et al., 2023 ^25^ | 12 | 148 | NR | 8 | 148 | 26 | 148 | 16 | 148 | 15 | 148 | 25 | 148 | NR | NR |
| Al-Waeli et al. 2022 ^26^ | NR | NR | NR | 28 | 475 | 1 | 475 | NR | NR | 18 | 475 | NR | NR | NR | NR |
| Asche et al., 2008 ^27^ | NR | NR | NR | 82 | 2326 | 35 | 2326 | 30 | 2326 | NR | NR | 30 | 2326 | NR | NR |
| Dandona et al., 1983 ^28^ | NR | NR | NR | NR | NR | 11 | 54 | NR | NR | NR | NR | NR | NR | NR | NR |
| Das et al., 2021 ^29^ | NR | NR | NR | NR | NR | 17 | 5695 | 14 | 5695 | NR | NR | 18 | 5695 | NR | NR |
| De Jong et al., 2016 ^30^ | 141 | 2490 | NR | 57 | 2490 | 369 | 2490 | NR | NR | 91 | 2490 | 159 | 2490 | 37 | 2490 |
| Florez et al., 2010 ^31^ | NR | NR | NR | 128 | 360 | 223 | 360 | 76 | 360 | 127 | 360 | 171 | 360 | NR | NR |
| Huang et al., 2015 ^21^ | 82 | 415 | digestive disturbance | 135 | 415 | 92 | 415 | 13 | 415 | 144 | 415 | 60 | 415 | NR | NR |
| Kim et al., 2012 ^22^ | 24 | 3556 | NR | 3 | 3556 | 37 | 3556 | NR | NR | 35 | 3556 | 22 | 3556 | 5 | 3556 |
| Levy et al., 2010 ^23^ | NR | NR | NR | 1 | 35 | 5 | 35 | NR | NR | NR | NR | 2 | 35 | NR | NR |
| Malik et al., 2023 ^32^ | NR | NR | NR | 0 | 62 | 2 | 62 | 3 | 62 | NR | NR | 3 | 62 | 1 | 62 |
| Memon et al., 2022 ^33^ | NR | NR | NR | NR | NR | 21 | 100 | 15 | 100 | NR | NR | 31 | 100 | NR | NR |
| Mishra et al., 2021 ^34^ | NR | NR | NR | NR | NR | 1 | 120 | 1 | 120 | NR | NR | NR | NR | NR | NR |
| Okayasu et al., 2012 ^35^ | 3 | 101 | diarrhea | NR | NR | 27 | 101 | 0 | 101 | NR | NR | 0 | 101 | NR | NR |
| Raičević et al., 2023 ^36^ | 5 | 330 | NR | 6 | 330 | 17 | 330 | NR | NR | 9 | 330 | 11 | 330 | NR | NR |
| Riyaz et al., 2014 ^37^ | NR | NR | NR | NR | NR | 9 | 50 | NR | NR | NR | NR | 4 | 50 | NR | NR |
| Sadeeqa et al., 2019 ^38^ | NR | NR | NR | 28 | 104 | 11 | 104 | 3 | 104 | 34 | 104 | 7 | 104 | 43 | 104 |
| Strojek et al., 2016 ^39^ | NR | NR | NR | 233 | 4737 | 259 | 4737 | 12 | 4737 | 15 | 4737 | 261 | 4737 | 1 | 4737 |
| Sumitani et al., 2012 ^40^ | 0 | 23 | NR | 0 | 23 | 6 | 23 | NR | NR | NR | NR | 0 | 23 | 0 | 23 |
| Umamaheswaran et al., 2015 ^41^ | NR | NR | NR | 19 | 122 | 2 | 122 | NR | NR | NR | NR | 5 | 122 | NR | NR |

Table 2. Risk of bias assessment.

|  | **SELECTION** | | | | **COMPARABILITY** | **OUTCOME** | | | **Total quality score** |
| --- | --- | --- | --- | --- | --- | --- | --- | --- | --- |
| **Reference** | **Representativeness of the exposed cohort** | **Selection of the non-exposed cohort** | **Ascertainment of exposure** | **Demonstration that outcome of interest was not present at start of study** | **Comparability of cohorts on the basis of the design or analysis** | **Assessment of outcome** | **Follow-up long enough for outcomes to occur** | **Adequacy of follow up of cohorts** |  |
| Aladhab et al., 2023 ^24^ | 1 | 1 | 1 | 1 | 1 | 0 | 0 | 0 | 5 |
| Alibrahim et al., 2023 ^25^ | 1 | 1 | 1 | 1 | 1 | 0 | 0 | 1 | 6 |
| Al-Waeli et al. 2022 ^26^ | 1 | 1 | 1 | 1 | 1 | 0 | 0 | 0 | 5 |
| Asche et al., 2008 ^27^ | 1 | 1 | 1 | 0 | 1 | 1 | 1 | 1 | 7 |
| Dandona et al., 1983 ^28^ | 1 | 1 | 1 | 0 | 1 | 0 | 0 | 0 | 4 |
| Das et al., 2021 ^29^ | 1 | 1 | 1 | 1 | 1 | 0 | 1 | 0 | 6 |
| De Jong et al., 2016 ^30^ | 1 | 1 | 1 | 1 | 1 | 1 | 1 | 0 | 7 |
| Florez et al., 2010 ^31^ | 1 | 1 | 1 | 1 | 1 | 1 | 1 | 0 | 7 |
| Huang et al., 2015 ^21^ | 1 | 1 | 1 | 1 | 1 | 1 | 1 | 0 | 7 |
| Kim et al., 2012 ^22^ | 1 | 1 | 1 | 1 | 1 | 1 | 1 | 1 | 8 |
| Levy et al., 2010 ^23^ | 1 | 1 | 1 | 1 | 1 | 1 | 1 | 0 | 7 |
| Malik et al., 2023 ^32^ | 0 | 1 | 1 | 1 | 1 | 1 | 1 | 0 | 6 |
| Memon et al., 2022 ^33^ | 1 | 1 | 0 | 1 | 1 | 0 | 0 | 0 | 4 |
| Mishra et al., 2021 ^34^ | 1 | 1 | 0 | 1 | 0 | 0 | 0 | 0 | 3 |
| Okayasu et al., 2012 ^35^ | 1 | 1 | 1 | 0 | 1 | 1 | 1 | 0 | 6 |
| Raičević et al., 2023 ^36^ | 1 | 1 | 0 | 1 | 1 | 0 | 0 | 0 | 4 |
| Riyaz et al., 2014 ^37^ | 1 | 1 | 1 | 1 | 1 | 1 | 1 | 0 | 7 |
| Sadeeqa et al., 2019 ^38^ | 1 | 1 | 1 | 1 | 1 | 1 | 1 | 0 | 7 |
| Strojek et al., 2016 ^39^ | 1 | 1 | 1 | 1 | 1 | 1 | 1 | 0 | 7 |
| Sumitani et al., 2012 ^40^ | 1 | 1 | 1 | 1 | 1 | 1 | 1 | 0 | 7 |
| Umamaheswaran et al., 2015 ^41^ | 1 | 1 | 1 | 1 | 1 | 1 | 1 | 1 | 8 |
